# Understanding *Zhongyong* Using a *Zhongyong* Approach: Re-examining the Non-linear Relationship Between Creativity and the Confucian Doctrine of the Mean

**DOI:** 10.3389/fpsyg.2022.903411

**Published:** 2022-06-15

**Authors:** Ruixiang Gao, Shiqi Huang, Yujie Yao, Xiaoqin Liu, Yujun Zhou, Shijia Zhang, Shaohua Cai, Huang Zuo, Zehui Zhan, Lei Mo

**Affiliations:** ^1^Philosophy and Social Science Laboratory of Reading and Development in Children and Adolescents (South China Normal University), Ministry of Education, Guangzhou, China; ^2^School of Psychology, South China Normal University, Guangzhou, China; ^3^School of Human-Environment Studies, Kyushu University, Fukuoka, Japan; ^4^School of Foreign Studies, South China Normal University, Guangzhou, China; ^5^School of Information Technology in Education, South China Normal University, Guangzhou, China; ^6^Center for Teacher Development, South China Normal University, Guangzhou, China; ^7^Institution for Teachers' Professional Ethics and Virtues Building (South China Normal University), Ministry of Education, Guangzhou, China

**Keywords:** *Zhongyong*, traditional Chinese culture, creativity, every coin has two sides, united contradiction perspective, too much of a good thing, implicit association test

## Abstract

*Zhongyong*, a central theme of Confucian thought, refers to the “doctrine of the mean,” or the idea that moderation in all things is the optimal path. Despite considerable interest in the relationship between *zhongyong* and creativity, especially in China, studies of this relationship have not yielded consistent results. Based on a review of the literature, we hypothesized that this inconsistency arises from the dual nature of *zhongyong* itself, which has both a positive side, promoting creativity, and a negative side, inhibiting creativity. We also hypothesized that the negative side of *zhongyong* takes the form of excessive *zhongyong*. Indeed, the observations that every coin has two sides and that too much of a good thing is as bad as too little are core principles of *zhongyong* in traditional Chinese culture. To test these hypotheses, we conducted two empirical studies (measuring explicit and implicit *zhongyong* personality, respectively) to examine the relationships between positive and negative *zhongyong* and creativity (measured in terms of creative personality, divergent thinking, and convergent thinking). The results of both studies revealed an interaction between positive *zhongyong* and negative *zhongyong*, indicating that only a moderate level of *zhongyong* is conducive to creativity; both deficiency and excess are harmful. We discuss the implications of these results, suggesting that a *zhongyong* approach can help to clarify non-linear relationships between things, and recommending to re-assess the creativity of Chinese culture from a neutral and objective outlook. This paper deepens understanding of *zhongyong* and offers clear insights into creativity from an in-depth cultural perspective.

## Introduction

The evolution of humankind is a story of creativity. From the creative use and invention of tools to solve survival problems to the use of innovative strategies to obtain developmental advantages, human activities have always been characterized by creativity (Li, 2017). In the twenty-first century, with the rapid growth of material civilization, governments worldwide are attaching increasing importance to the stimulation and cultivation of creativity on a national level. China, one of the world's four oldest civilizations, is no exception. However, despite its early progress in scientific discovery, China was swiftly overtaken by the West in terms of modern scientific and industrial development (the “Joseph Needham puzzle”). Similarly, the “Qian Xuesen's question”—“why can't our universities cultivate such outstanding talent?”—reflects many Chinese people's concern about a lack of creativity in modern China. In recent years, cross-cultural studies have added weight to this concern. For example, Hu et al. (2004) sampled 2,277 Chinese and British teenagers aged 11 to 18 and found that the British teenagers showed significantly higher levels of scientific creativity than the Chinese teenagers did. Zha et al. (2006) studied 55 American and 56 Chinese doctoral candidates and found that although the Chinese participants had significantly better mathematical skills than the American participants, they scored significantly lower for creative potential. The authors argued that the latter result was related to China's highly collectivist culture. Similar discoveries have been made regarding artistic creativity (Niu and Sternberg, 2001; Yi et al., 2013). Together, these findings suggest that cultural differences affect creative performance.

To study the influence of Chinese culture on creativity, it is necessary to study *zhongyong* (“中庸”). *Zhongyong*, the doctrine of the golden mean, is a core concept in traditional Chinese culture, which originated in Confucian thought. *Zhongyong* refers to the principle of the desirable middle between two extremes. It was once regarded as a virtue of the highest order and shaped Chinese people's values, beliefs, and mindsets. However, since ancient times, the connotations of the term *zhongyong* have shifted. To many modern Chinese people, *zhongyong* is a derogatory term, synonymous with “having no independent view and fixed position” (折衷主义), “avoiding speaking up even when necessary” (不出头), “seeking peace without principles” (和稀泥), “always saying yes just to avoid offending” (老好人), and “being equivocal” (模棱两可). Many now believe that following the principle of *zhongyong* will lead to “mediocrity” (平庸), “ordinariness” (庸碌), and “the accomplishment of nothing” (无所作为) (Liu, 2019). The shift in understanding of *zhongyong* between ancient and modern times has led to inconsistencies in the conceptualization, operational definition, and measurement of *zhongyong*, which in turn have led to inconsistencies in research findings. To date, studies exploring the relationship between *zhongyong* and creativity have not yielded consistent results: some have found a significant positive correlation, some a significant negative correlation, and others no significant correlation.

We argue that the conceptualization of *zhongyong* in previous studies has not itself followed the doctrine of *zhongyong*, which holds that we should treat things dialectically. As the saying goes, “every coin has two sides.” Thus *zhongyong* itself cannot be entirely positive; it must also have a negative side. *Zhongyong* also teaches that “too much of something is as bad as too little,” suggesting that its influence on creativity may not be linear. We therefore re-conceptualize *zhongyong* into two categories, positive *zhongyong* and negative *zhongyong*, and conceptualize the latter as an excessive manifestation of the former. To control for social desirability bias in the results, we also adopt for the first time an implicit association test (IAT) to measure *zhongyong*. In the current paper, we present two empirical studies (which measure explicit and implicit *zhongyong* identity, respectively) examining the relationship between positive and negative *zhongyong* and creativity (which is measured as creative personality, divergent thinking, and convergent thinking).

## Analytical Framework

### Connotations of *Zhongyong* at the National and Global Levels

Although the Chinese word *zhongyong* (中庸) was coined by Confucius, the concept of *zhongyong* existed long before Confucius; it can be traced back to the Five *Classics* (五经) of ancient China. The core meaning of the word *zhongyong* lies in its first Chinese character, *zhong* (中), which refers to the broad idea of “correctness.”

At first, *zhongyong* was seen mainly as the moral requirement for a ruler to be fair and just in political management. Of the three virtues advocated in “The Great Plan” of the *Book of Documents* (《尚书·洪范》), correctness/straightforwardness (正直) was listed in the first place. “The Announcement About Drunkenness” in the *Book of Documents* (《尚书·酒诰》) stated that correctness is achieved “when you can maintain a constant, watchful examination of yourselves, and your conduct is in accordance with correct virtue” (克永观省, 作稽中德). This was the first time that correctness had been explicitly deemed a virtue of rectitude and righteousness. Only when a ruler is honest, sincere, and upright without partiality can he govern well (Legge, 1879).

The full word *zhongyong* first appeared in “Yong Ye” of *The Analects* (《论语·雍也》), in which the Master said: “Perfect is the virtue which is according to the Constant Mean! Rare for a long time has been its practice among the people” (中庸之为德也, 其至矣乎！民鲜久矣). Although Confucius did not define *zhongyong* directly, he exemplified the doctrine with reference to “five excellent things” (五美): “when the person in authority is beneficent without great expenditure; when he lays tasks on the people without their repining; when he pursues what he desires without being covetous; when he maintains a dignified ease without being proud; when he is majestic without being fierce” (君子惠而不费, 劳而不怨, 欲而不贪, 泰而不骄, 威而不猛; in “Yao Yue” of *The Analects* (《论语·尧曰》) (Legge, 1861). Confucius transformed *zhongyong* from being a moral requirement for rulers to being a moral requirement for everyone in society who sought to better themselves (君子), defining it as behavior that conforms to the requirements of propriety (礼).

Confucius' disciples later compiled “The State of Equilibrium and Harmony” (《中庸》) as a chapter of *The Classic of Rites* (《礼记》), explaining that, “While there are no stirrings of pleasure, anger, sorrow, or joy, the mind may be said to be in the state of Equilibrium. When those feelings have been stirred, and they act in their due degree, there ensues what may be called the state of Harmony. This Equilibrium is the great root from which grow all the human actings in the world, and this Harmony is the universal path which they all should pursue” (喜怨哀乐之未发, 谓之中; 发而皆中节, 谓之和。中也者, 天下之大本也。和也者, 天下之达道也). In other words, *zhongyong* is the achievement of equilibrium and harmony (中和) by holding a middle ground (执中). This means that people's feelings, desires, thoughts, and behaviors should be controlled within a reasonable range, an idea that became the broadest understanding of *zhongyong* (Legge, 1885).

Cheng Yi, a neo-Confucianist of the Northern Song dynasty (A.D. 960–1127), wrote: “What is not extreme is called ‘*zhong*', and what is not changing is called ‘*yong*”' (不偏之谓中, 不易之谓; Zhu, 2012). Master Zhu Xi of the Southern Song dynasty (A. D. 1127–1279) explained that “‘*Zhong*' means impartial and moderate, and ‘*yong*' means constant” (中者, 不偏不倚、无过不及之名。庸, 平常也) in *Variorum of the Four Books* (《四񎙨章句集注》; Zhu, 2012). *Zhong* (中), therefore, has the meanings of moderation, appropriateness, and propriety (which is the valued standard in Confucianism), while “*yong*” (庸) means holding such a state and remaining unchanged. Therefore, *zhongyong* can also be interpreted as continuously holding the middle ground or always adhering to the appropriate rules of conduct. This is considered to reflect the morality of a superior person (君子).

Over time, with the integration of Confucianism, Taoism, and Buddhism, the Confucian concept of *zhongyong* gained new connotations.

The call for moderation in *zhongyong* echoed the concept of the unity of opposites in Taoism, and the connotations of *zhongyong* were expanded to include a perspective on the world in addition to the original moral aspect. Taoism, with its well-known yin–yang diagram (太极图), has an even more profound history than Confucianism, although it was never declared to be the state ideology of ancient China by the emperor. Laozi, the teacher of Confucius, who is said to have composed the *Dao De Jing* (《道德经》), explained the *dao* (道), the law of mutual transformation between yin and yang (阴阳转化), as follows: “it is that existence and non-existence give birth the one to (the idea of) the other; that difficulty and ease produce the one (the idea of) the other; that length and shortness fashion out the one the figure of the other; that (the ideas of) height and lowness arise from the contrast of the one with the other; that the musical notes and tones become harmonious through the relation of one with another; and that being before and behind give the idea of one following another” (有无相生, 难易相成, 长短相较, 高下相倾, 音声相和, ‘O@‘Š) (Legge, 1891). This idea can be traced back to the *Book of Changes* (《易经》), particularly its later supplementary chapter “The Great Treatise” (《系辞》), written by Confucius, which states: “The successive movement of the inactive and active operations constitutes what is called the course (of things). That which ensues as the result (of their movement) is goodness; that which shows it in its completeness is the natures (of men and things)” (一阴一阳之谓道, 继之者善也, 成之者性也) (Legge, 1899). This shows that Confucianism inherited the outlook of dialectical unification from Taoism and folded it into the *zhongyong* moral principle. A *zhongyong* person, therefore, was expected to carefully consider every aspect of a problem when dealing with it and to adopt an objective, neutral standpoint when reconciling different opinions. In “The State of Equilibrium and Harmony” in *The Classic of Rites* (《礼记·中庸》), Confucius provided an example of such a person: “There was Shun: He indeed was greatly wise! Shun loved to question others, and to study their words, though they might be shallow. He concealed what was bad in them and displayed what was good. He took hold of their two extremes, determined the Mean, and employed it in his government of the people. It was by this that he was Shun!” (舜其大知也与！舜好问而好察迩言, 隐恶而扬善, 执其两端, 用其中于民, 其斯以为舜乎！) (Legge, 1885).

Conversely, the attitude of aloofness in Buddhism lent to *zhongyong* an apparently negative aspect of unprincipled eclecticism that was against Confucius' original intention. In Buddhist thought, escaping the material world is the ultimate goal. According to the “Three Marks of Existence” (Sanskrit *ti-lakkhana*), the basic characteristics of the world are impermanency and constant change (Sanskrit *anicca*). The failure to recognize this, and the practice of clinging to things as if they were permanent, results in dissatisfaction, discomfort, anxiety, frustration, sorrow, pain, suffering, and misery (Sanskrit *duḥkha*). The path to Buddhahood is through a careful examination of the constantly changing constituents of a person or an object, by which the practitioner gradually comes to the conclusion that there is no abiding substance in the existence of human beings, other forms of life (“no-self,” Sanskrit *anatta*), or non-living things (emptiness, Sanskrit *Sunyatā*). The practitioner ultimately reaches a state of liberation from the cycles of rebirth and transcends suffering (*nirvana*; Anderson, 2013). Buddhism's pessimistic outlook on life may have served as comfort for Confucian students who were unsuccessful in their official careers and who either intentionally or unintentionally misinterpreted *zhongyong* as compromise, indifference, or equivocation, even though this deviated from the moral principles promoted by Confucianism. In “Zi Lu” of *The Analects* (《论语·子路》), the Master said: “The superior man is affable, but not adulatory; the mean man is adulatory, but not affable” (君子和而不同, 小人同而不和) (Legge, 1861). According to “Jin Xin II” of *The Works of Mencius* (《孟子·尽心下》), Confucius called these hypocrites *xiangyuan* (乡原), saying that those who blurred the line between right and wrong for the sake of patching up a quarrel were thieves of virtue (德之贼) (Legge, 1985). However, this incorrect interpretation became increasingly popular and is even more popular today.

**Figure 1:**
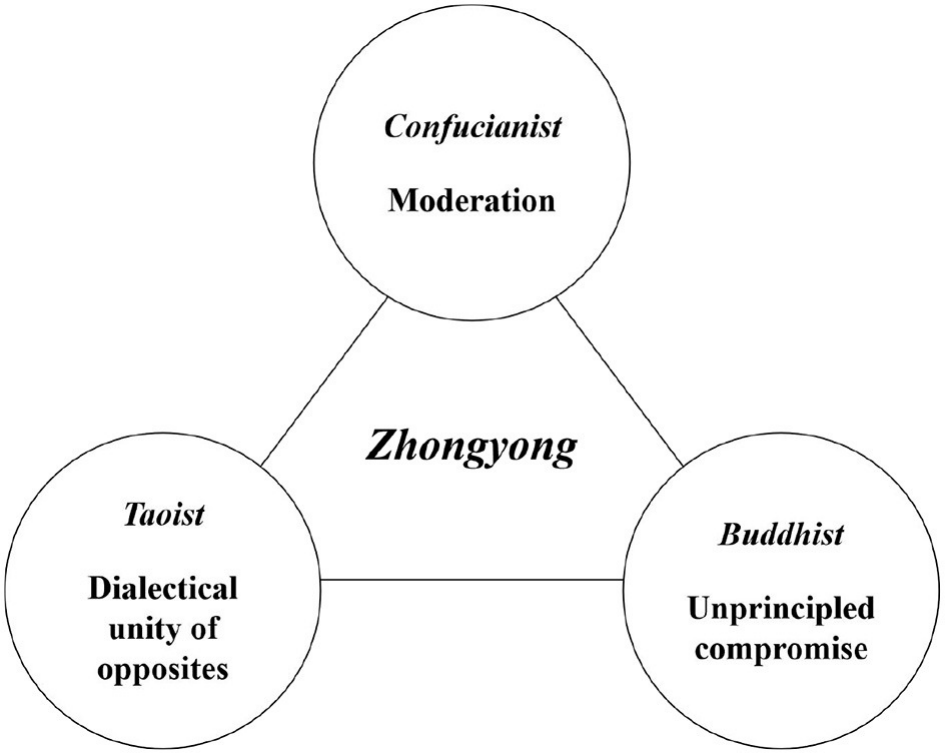
Diagram showing the tripartite connotations of *zhongyong* in present-day China.

Today, *zhongyong* has tripartite connotations (see Figure 1): (a) moderation, its original meaning, from Confucianism; (b) the dialectical unity of opposites, from Taoism; and (c) a tendency to compromise, from Buddhism (with the specifically derogatory implication of unprincipled compromise). As the tradition of Confucian propriety (礼) has gradually died out in modern China since the Attack on the Four Olds (破四旧, i.e., old thoughts, old culture, old habits, and old customs) in the Great Cultural Revolution, Confucianist moderation has become increasingly de-emphasized. This is partly attributable to its misinterpretation as unprincipled compromise. Of the three sets of connotations of *zhongyong*, the Taoist conception of *zhongyong* as dialectical thinking is now the most common.

Pang, 1980, 2000 argued that the dialectical unity of opposites provides methodological guidance for achieving moderation in daily practice. Specifically, as a way of thinking about things, *zhongyong* involves two opposite sides of an object, A and B (一体两面, “one body and two sides”). A and B mutually generate and restrict each other (相生相克), representing the contradictory relationship of the unity of opposites. Pang identified four forms of *zhongyong*: (1) “complementing A with B,” or using the opposite B to supplement A's deficiency; (2) “A but not A,” or removing the negative aspects of A to prevent A from becoming extreme; (3) “neither A nor B,” or being impartial and avoiding too much or too little of anything; and (4) “both A and B,” or the combination (or dynamic balance) at different stages and on different occasions.

The ideology of *zhongyong* is not unique to China. The ancient Greek philosopher Aristotle proposed a doctrine of the golden mean that was identical to the Confucian *zhongyong*. Aristotle argued that virtuous habits of action were often an intermediate condition between two extremes, one of excess and the other of deficiency, and that too much or too little was always wrong. He stated: “virtue must have the quality of aiming at the intermediate. I mean moral virtue; for it is this that is concerned with passions and actions, and in these there is excess, defect, and the intermediate. For instance, both fear and confidence and appetite and anger and pity and in general pleasure and pain may be felt both too much and too little, and in both cases not well; but to feel them at the right times, with reference to the right objects, toward the right people, with the right motive, and in the right way, is what is both intermediate and best, and this is characteristic of virtue” (Aristotle, 1999, p. 27). The philosophy of dialectical materialism, developed by the founders of Communist philosophy Karl Marx and Frederick Engels, conveyed a world outlook similar to that of Taoism and became widespread in modern China. “The central idea of dialectics is the unity and struggle of opposites, that is, contradictory tendencies that are tied together and cause things to change and develop … [T]hey (Marx and Engels) did not start from scratch. They borrowed ideas from a long history of dialectical thought that dates back at least 25 centuries in Europe and was developed independently in China and India.” The Chinese sources of this dialectical thought were the *Book of Changes* (《易经》), the *Dao De Jing* (《道德经》), and *Mohism* (《墨子》; International Communist Workers Party (ICWP)., 2013). However, although the West developed philosophies similar to *zhongyong*, in Chinese culture this philosophy became deeply embedded in value systems, ways of thinking, and daily conduct. This may be because the Chinese “virtue-oriented” educational model focuses more on perfecting the self *via* social ideology, while the Western model emphasizes exploring the outside world (Li, 2012; Gao et al., 2022).

### Measurement of *Zhongyong* in Previous Research

The systematic psychological study of *zhongyong* began in the late 1990s, with research by Yang and her colleagues. These scholars attempted for the first time to conceptualize *zhongyong* in psychological research, proposing that it was a practical thinking system that people used to decide how to choose, execute, and correct specific action plans when dealing with routine actions. Their system consisted of eight main sub-constructs: the unity of humans and nature (天人合一), bipolar thinking (两极思维), consequential thinking (后果思维), waiting to see what happens (静观其变), not going to extremes (不走极端), considering the overall situation (顾全大局), being reasonable (合情合理), and retreating to advance (以退为进). Based on this conceptualization, they developed a Zhong-Yong Practice Cognition Scale containing 16 forced-choice items (Yang and Chiu, 1997). Since then, Yang has continued to develop the psychological conceptualization of *zhongyong* into a cultural meaning system that can enter into dialog with Western social psychology (Yang, 2008). Yang constructed a conceptualization diagram of the *zhongyong* practice cognition system (CDZPCS) as a blueprint for the study of the role of *zhongyong* in Chinese people's lives (Yang, 2009). She also provided a research roadmap based on this blueprint (Yang, 2010) and applied traditional psychological research methods to test the construct validity of the CDZPCS (Yang and Lin, 2012; Yang et al., 2014).

However, Yang's system was argued to be overcomplicated and was later revised by her collaborators. Chiu (2000) concretized *zhongyong* thinking at the level of action and divided it into three dimensions: “taking harmony as the action goal” (以“和'为行动目标), “recognizing the complex interrelationships between things” (认清事物复杂的相互关系), and “carrying out actions with reference to a middle ground” (以“执中”开展行动). Based on this reconceptualization, the original scale was reduced to 14 items and then tested in five Chinese communities. However, this new scale was questioned in relation to its quantitative methods, reliability, and validity (Wu and Lin, 2005; Huang et al., 2012). Huang et al. (2012) revised the 16-item scale into a 9-item Zhong-Yong Belief–Value Scale (ZYBV), including two dimensions of self-convergence (自我收敛) and vision elevation (拔高视野). This version has been widely used in research. Other researchers have focused on the basic meaning of *zhongyong*, that is, “master the extremes, but deploy the mean” (执两端而允中). In other words, when dealing with a controversial issue, one should consider a range of perspectives in detail and make decisions that take into account both the overall situation and the self. Huang et al. (2012) also established the Zhong-Yong Thinking Style Scale (ZYTS), which featured three dimensions: “multi-dimensional thinking” (多方思考), “holism” (整合性), and “harmoniousness” (和谐性). This scale is currently one of the most widely used tools for measuring *zhongyong* (Wu and Lin, 2005). Du and Yao (2015) argued that the scales designed by Chiu (2000) and Wu and Lin (2005) were based on ideas of *zhongyong* extracted from the theoretical literature and therefore belonged to “classic *zhongyong*.” They argued that because the concept of *zhongyong* has changed over time, these scales did not measure *zhongyong* as it was perceived and applied by Chinese people today. They therefore investigated Chinese enterprise employees' perceptions of *zhongyong* using an open questionnaire, then proposed four dimensions of *zhongyong*: “mean and congruence” (执中一致), “personal cultivation” (慎独自修), “no ambition” (消极成就), and “passive avoidance” (消极规避). They identified mean and congruence as the core and went on to investigate the relationship between mean and congruence and collectivism.

In addition to using questionnaires for the static measurement of *zhongyong* as a relatively stable personality trait or thinking habit, some researchers have used other research paradigms, such as situational question priming, to investigate *zhongyong*. Studies have shown that the thinking of East Asians is more context-dependent than that of Westerners (e.g., Ji et al., 2000), and adaptability is also an important feature of *zhongyong*. Based on the four forms of *zhongyong* proposed by Pang (1980), Zhou et al. (2019); Zhang et al. (2020) summarized two main forms of *zhongyong* thinking: eclectic thinking and integrated thinking. “Eclectic thinking” refers to the cognition of individuals who fail to recognize the essential problems underlying contradictory information, leading them to compromise to alleviate superficial or temporary contradictions, which ultimately makes it difficult to solve the problems. This type of thinking occurs in two situations: either a problem is beyond the ability of the solver or the solver avoids making cognitive effort due to laziness. “Integrated thinking” refers to the ability to understand the essence of a problem and synthesize seemingly contradictory information to solve the problem. Integrated thinking takes place at a higher level than eclectic thinking. On this basis, Zhou et al. (2019); Zhang et al. (2020) pioneered a causal experimental approach to studying *zhongyong* by developing situational problem materials to prime eclectic thinking and integrated thinking. Studies have incorporated Western ideas about paradoxical thinking (Miron-Spektor et al., 2011; Leung et al., 2018), a concept similar to *zhongyong* thinking.

### Relationship Between Zhongyong and Creativity

Creativity is a multidimensional construct that captures the ability of an individual to solve a problem in a novel way (Jiao et al., 2017; Li et al., 2017; Lin et al., 2018). The measurement of creativity typically reflects one of two definitions of creativity: the first is as a personality trait, and is usually measured by self-report questionnaires; while the second is as a set of cognitive capabilities (e.g., divergent thinking and convergent thinking), and is usually measured by ability tests with differing degrees of difficulty. *Zhongyong*, as a cultural factor that impacts creativity, has received increasing research interest; however, studies examining the relationship between *zhongyong* and creativity have not yielded consistent results. Several studies (Zhang and Gu, 2015; Yang and Zhang, 2018) have found that the *zhongyong* thinking of employees in enterprises is positively correlated with their innovative behaviors. *Zhongyong* thinking has also been shown to positively predict individual (Liao and Dong, 2015) and team (Chen et al., 2018) innovative behaviors. Some studies, however, have found that *zhongyong* might hinder innovation. For example, a negative correlation has been found between the ZYTS scores of Chinese art students and their creative personality scores (Liu et al., 2015). Other studies have found that the relationship between the two is not linear. For example, Yao et al. (2010) found that scores on the ZYTS scale moderated the relationship between self-evaluated creativity and leader-evaluated innovative behaviors: there was no significant correlation between the two in the high-*zhongyong* group of participants, but there was a significant correlation in the low-*zhongyong* group, implying that *zhongyong* hindered the transformation of creative ideas into innovative action. Du et al. (2018) found that *zhongyong* value orientation promoted incremental innovation but inhibited radical innovation. All of these studies used questionnaires to measure creativity and *zhongyong* thinking. Most of the studies that found negative or no correlations between *zhongyong* and creativity (e.g., Yao et al., 2010; Liu et al., 2015) used the ZYBV scale, while most of those that found a positive correlation (e.g., Liao and Dong, 2015; Zhang and Gu, 2015) used the ZYTS scale. We suggest that this is because the ZYBV scale uses a forced-choice method, making it less prone to social desirability bias.

Other researchers have used non-questionnaire methods to measure creativity. Chang and Yang (2014) used participants' performance on a redundant-target detection task as indicators of creativity, finding that high *zhongyong* thinkers, as identified using the ZYTS scale, processed information more efficiently and in a more integrated fashion than low *zhongyong* thinkers. Similarly, Wang et al. (2013) used participants' eye tracking performance when viewing banner advertisements as indicators of creativity, finding that high-*zhongyong* thinkers, as identified using the ZYBV scale, exhibited a more efficient and flexible perceptual style when switching between global processing and local processing. Huang et al. (2014) further found that when primed with emotional words, the high-*zhongyong* group of participants, as selected using the ZYBV scale, showed significantly more global precedence (i.e., stepping back to see the whole picture). However, when the priming was absent, there was no reliable relationship between *zhongyong* and global processing speed. This implied that *zhongyong* served as an emotional regulator that affected individuals' cognitive processing strategies, affirming Confucius' statement that “while there are no stirrings of pleasure, anger, sorrow, or joy, the mind may be said to be in the state of Equilibrium” (喜怨哀乐之未发, 谓之中). Recently, researchers used participants' performance on a divergent thinking test (the Alternative Uses Task, AUT), a convergent thinking test (the Remote Associates Test, RAT), and insight problem-solving tests (Chinese idiom puzzle problems, brain-teaser problems, and market investment problems) as indicators of creativity and found that there was no significant correlation between scores on the ZYTS and ZYBV scales and these indicators (Zhang et al., 2020). When the participants were primed with a *zhongyong* conditional problem, those primed with integrated thinking performed better in the RAT and the market investment problems than those primed with eclectic thinking and the control group (Zhang et al., 2020; Zhou et al., 2021). The improved RAT performance was supported by EEG data (Zhou et al., 2019), suggesting that the RAT and the integrated thinking priming tasks involve the same neural mechanism. Researchers examining the Western counterpart of *zhongyong*, paradoxical thinking, have typically viewed it as a mental template for approaching contradictory yet interrelated elements to enable change and gain new insights (Gordon, 1961; Fletcher and Olwyler, 1997; Lewis, 2000; Martin, 2009; Ingram et al., 2016; Miron-Spektor and Erez, 2017). Miron-Spektor et al. (2011) found that priming paradoxical frames promoted participants' creative thinking. They argued that this was because the sense of conflict caused by the paradoxical relationship led to a willingness to embrace different perspectives and to integrate these different perspectives by generating new linkages among them, thus promoting creativity. However, Leung et al. (2018) found that people who endorsed a middle-ground approach were less likely to find integrative solutions and thus received fewer of the creative benefits of paradoxical frames.

Based on the above review, it is evident that there is both strong support for and considerable doubt about the idea that *zhongyong* promotes creativity. The main argument supporting the promotion of creativity is that a person who practices integrative thinking is better at viewing problems from a global perspective and adopting flexible strategies to integrate different or even contradictory opinions, and thus is more able to produce new ideas that bridge differences and achieve harmony [see Chang et al., 2014 for a detailed review of the relationship between *zhongyong* and the six dimensions of wisdom, as per Grossmann et al. (2010, 2013): compromise, recognition of the limits of knowledge, flexibility, perspective-taking, recognition of change, and resolution of conflict]. The primary argument in favor of *zhongyong* hindering creativity is that a *zhongyong* person with middle-ground, eclectic thinking tends to avoid conflict and seek interpersonal harmony, thus compromising easily without challenging authority or social norms. This is a typical argument for the view that collectivist cultures inhibit creativity (Hofstede et al., 2010). While researchers have been puzzled by these seemingly contradictory results, we believe that the results precisely reflect the essence of *zhongyong*. The dialectical thought of *zhongyong* tells us that there is no absolute good or bad and that everything has a positive and a negative side. *Zhongyong* itself is no exception. The positive side of *zhongyong*, integrative thinking, is conducive to creativity, while the negative side, eclecticism, is harmful to creativity. The inconsistent results of previous studies have arisen from a confusion of positive *zhongyong* with negative *zhongyong*—simply reflecting the ability of *zhongyong* to unify contradictory things. With further analysis, we also argue that the pursuit of *zhongyong* is characterized by moderation, while the negative aspects of *zhongyong*, such as unprincipled compromise, arise from an excessive amount of *zhongyong*, indeed. For example, making a decision without considering different points of view is inadvisable, but if someone is *too* cautious and hesitant when combining viewpoints to make a decision, he/she is criticized for exercising eclectic thinking rather than praised as a dialectic thinker. Therefore, to understand the research results of *zhongyong*, we must therefore adopt *zhongyong* thinking.

### The Current Studies

To testify the above assumptions, we first distinguished between positive *zhongyong* and negative *zhongyong*. To define and describe the two, we used 12 Chinese idiomatic expressions, six for each. For positive *zhongyong*, the expressions were as follows: “taking the whole situation into account” (顾全大局), “cherishing peace and harmony” (ÒÔºÍÎª¹ó), “properly following rules for advancing and retreating” (进退有度), “knowing when to bend or stand upright” (能屈能伸), “leaving some leeway” (留有余地), and “being impartial” (不偏不倚). For negative *zhongyong*, the idiomatic expressions were as follows: “being worldly-wise to avoid getting into trouble” (明哲保身), “being a yes-man” (好好先生), “flattering both sides” (两面讨好), “swaying both ways” (左右逢源), “making concessions against one's will for a semblance of peace” (委曲求全), and “being untrustworthy” (生性圆滑). To select these 12 Chinese idioms, three experts in the authorship first brainstormed two initial lists of *zhongyong*-related expressions, one for positive *zhongyong* and the other for negative *zhongyong*. Then, another five experts were invited to supplement new items to the lists, which resulted in 18 expressions for positive *zhongyong* and 14 for negative *zhongyong*. Next, 95 university students were recruited to evaluate the representativeness, familiarity, and semantic valence (positive or negative) of these 32 expressions. Eventually, based on the evaluation, two experts in the authorship decided that six idioms for positive *zhongyong* and six for negative *zhongyong* were selected as the final expressions.

Based on these definitions, we conducted two empirical studies to examine the relationship between positive and negative *zhongyong* and creativity (creative personality, divergent thinking, and convergent thinking). As the descriptive terms for negative *zhongyong* were pejorative, in Study 2 we adopted an implicit association test (IAT) to minimize social desirability bias. The IAT, introduced by Greenwald et al. (1998), is a measure widely used in social psychology to detect attitudes and beliefs that people may not be willing or able to report. The instrument measures each participant's reaction time and accuracy rate, assuming that they are reflective of automatic associations between mental representations. As positive *zhongyong* and negative *zhongyong* might partially overlap because of our operational definitions, we expected to see an interaction between the two. We predicted that when a participant's level of negative *zhongyong* was low, which means that overall *zhongyong* had not reached an extreme level, positive *zhongyong* would positively predict creativity; that is, *zhongyong* is conducive to creativity as long as it is not excessive. However, when their level of negative *zhongyong* was high, which means that *zhongyong* overall had reached an extreme level, positive *zhongyong* would have either no correlation or a negative correlation with creativity; in other words, too much *zhongyong* is detrimental to creativity. If these two parts of hypothesis could be verified, we could reach a conclusion that only a moderate level of *zhongyong* is beneficial for creativity.

## Study 1

In Study 1, we developed and used a positive and negative *zhongyong* personality scale to explore the non-linear relationship between positive and negative *zhongyong* and creativity (creative personality, divergent thinking, and convergent thinking).

### Participants and Procedure

Before recruiting the participants, we used G^*^Power 3.1 to estimate the minimum sample size suitable for interaction analysis in a three-predictor regression model. According to Faul et al. (2007), when setting the effect size (*f*^2^) at 0.15 (medium level), the error probability (α) at 0.05 (as a common practice), the power (1 β) at 0.8 (as a common practice), the number of tested predictors at 1, and the total number of predictors at 3, the calculated minimum sample size was 55.

In reality, one hundred and fourteen undergraduate students (27 male, 87 female) aged between 18 and 22 years (*M* = 19.23, *SD* = 0.99) from two classes of a university-wide selective course took part in the study during a classroom-based session. None of the participants had experience of taking tests similar to those involved in our study. After they consented to participate, the students were asked to complete the AUT, the RAT, the creative personality scale, the ZYTS scale, the ZYBV scale, and our *zhongyong* personality scale (in that order) using their mobile telephones. The instruments were administered *via* Wenjuanxing, a Chinese online questionnaire survey platform. The total duration for all instruments was ~25 mins.

### Methods

#### Measuring Creativity

Three types of tasks were used to measure creativity: a Chinese version of the Williams Scale, an Alternative Uses Task (AUT, for measuring divergent thinking) drawn from the Torrance Tests of Creative Thinking, and a Chinese Remote Associates Test (RAT, for measuring convergent thinking test) that we compiled for the purposes of this study. The first instrument, the Chinese Williams Scale, was used to measure creative personality. It consists of four dimensions and 50 items (8 reverse-scored): 14 for curiosity, 13 for imagination, 12 for complexity, and 11 for risk-taking (Lin and Wang, 1999). In the current sample, the 3-point Likert scale we used had a Cronbach's α of 0.88. The second instrument, the AUT, was used to measure divergent thinking, or the ability to think of solutions to a problem from various angles. In this test, participants are given 5 mins and asked to list as many ways as possible that a common item (a cardboard box) could be used (Torrance, 1966). The participants' answers were scored according to three criteria, namely fluency, flexibility, and originality, with their overall score calculated as the mean of the three. This scale has already been shown to have high inter-rater reliability. In a random sample of 89 in another study we conducted, two coders achieved inter-rater reliability values (calculated using a Pearson correlation) of 0.99, 0.95, and 0.89 for each of the three components, respectively. In the current research (both Studies 1 and 2), the answers were scored by one of these two coders. The third instrument, the Chinese RAT, was used to measure convergent thinking, or the ability to apply established rules and logical reasoning to narrow down the possible solutions to a problem. Our instrument was a modified version of Wu and Chen (2017) instrument and contained 50 items suitable for college students. Each item comprised three clue characters, e.g., “原” (plain), “鞋” (shoe), and “野” (wild). The participants had 10 secs to come up with the answer, which was a target character, e.g., “草” (grass), that had a semantic connection with all three clues and created three actual two-character words, e.g., “草原” (grassland), “草鞋” (straw sandal), and “野草” (weed). The pass rate for this instrument was 0.23.

#### Measuring Zhongyong

Three scales were used to assess *zhongyong*: the 13-item ZYTS scale (Wu and Lin, 2005), the nine-item ZYBV scale (Huang et al., 2012), and the *zhongyong* personality scale that we compiled for the purposes of this study. The ZYTS scale used a 7-point Likert scale, with 7 indicating “extremely like me,” and had no reverse-scored items. The ZYBV scale presented the participants with two contradictory statements and prompted them to choose the one they agreed with. The participants then evaluated the degree to which they agreed with the statement on a 7-point Likert scale, with 7 indicating complete agreement. If the participant chose a non-*zhongyong* statement, the score of the corresponding item was reversed. These two scales were used as criteria in our study and had Cronbach's α values of 0.91 and 0.61, respectively. The *zhongyong* personality scale that we compiled was based on Gough (1979) Creative Personality Scale. This instrument consisted of an adjective checklist that the participants marked to indicate whether each adjective described them well. The list consisted of 32 adjectives, 6 positive *zhongyong* terms, 6 negative *zhongyong* terms (see section 2.4), and 20 terms not relevant to *zhongyong* (such as “hardworking” and “careful”) that were not used in this study. The participants scored between 0 and 6 for both positive and negative *zhongyong*.

### Results

Descriptive statistics and Pearson correlation coefficients for the participants' scores on the six tasks and scales of interest are shown in Table 1. Positive *zhongyong* had a positive and significant correlation with negative *zhongyong*, ZYTS, and ZYBY, while negative *zhongyong* had no significant correlation with ZYTS or ZYBY. This supported our argument that the instruments previously used to assess *zhongyong* have considered only its positive side, neglecting its negative side. Positive *zhongyong* had a positive and significant correlation with RAT, while negative *zhongyong* had a negative and significant correlation with creative personality. This supported our argument that *zhongyong* has two opposite sides, one promoting creativity and the other inhibiting it.

**Table 1:** Descriptive statistics and correlation coefficients for the six measures.

	***M***	***SD***	**Positive *zhongyong***	**Negative *zhongyong***	**ZYTS**	**ZYBV**	**Creative personality**	**AUT**	**RAT**
Positive *zhongyong*	5.04	1.24	1						
Negative *zhongyong*	2.60	1.61	0.27^**^	1					
ZYTS	5.71	0.76	0.45^**^	0.14	1				
ZYBV	5.18	0.69	0.39^**^	0.15	0.28^**^	1			
Creative personality	2.17	0.24	−0.12	−0.23^*^	0.14	−0.01	1		
AUT	23.70	9.70	−0.03	0.06	0.25^**^	−0.08	0.18	1	
RAT	11.67	3.57	0.24^**^	−0.06	0.17	0.30^**^	0.06	0.06	1

* *p < 0.05*,

** *p < 0.01*.

Table 2 displays the frequency distributions of positive *zhongyong* and negative *zhongyong*, dividing scores into two groups: [0,3] and [3,6]. We can see that the majority of the participants scored high on positive *zhongyong* and low on negative *zhongyong*. Only one participant scored high on negative *zhongyong* and low on positive *zhongyong*, which suggests that someone with a negative zhongyong personality must first have developed a positive zhongyong personality, and therefore, supports our argument that negative *zhongyong* is an excessive form of *zhongyong*.

**Table 2:** Cross-tabulation of frequency distributions for positive and negative *zhongyong*.

		**Negative** ***zhongyong***		**Total**
		**[0,3]**	**[3,6]**	
Positive *zhongyong*	[0,3]	14	1	15
	[3,6]	69	30	99
Total	83	31	114

**Figure 2:**
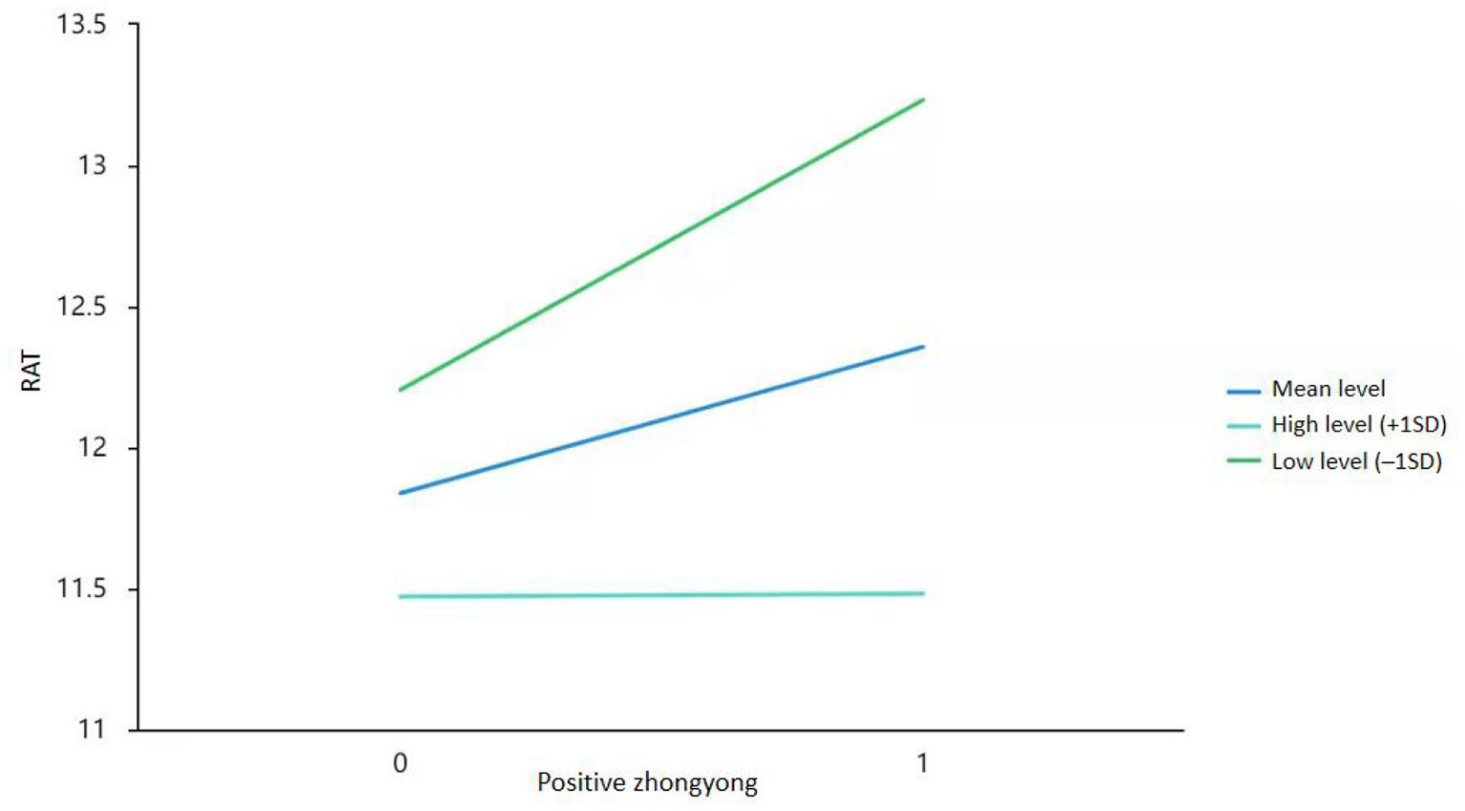
The moderating effect of negative *zhongyong* on the correlation between positive *zhongyong* and creativity.

As positive *zhongyong* and negative *zhongyong* are positively correlated, we expected to see an interaction in their effects on creativity. Specifically, we expected negative *zhongyong* to moderate the correlation between positive *zhongyong* and creativity. Therefore, after controlling common method bias by Harman's single-factor test (the percentage of variance for the first common factor was 28.87% <40%) and centralizing the data, we computed three regression models, one each for creative personality, the AUT, and the RAT, using positive *zhongyong*, negative *zhongyong*, and their product term as independent variables (see Table 3). However, we found only a marginally significant (*p* = 0.07) interaction in the regression model for the RAT. We then conducted a simple slope test (see Figure 2) to examine the moderating effect of negative *zhongyong* and found that the resulting tendency was in line with our expectation that at low levels of negative *zhongyong*, positive *zhongyong* would positively predict creativity, but at high levels of negative *zhongyong*, positive *zhongyong* would negatively predict or fail to predict creativity.

**Table 3:** Regression models for creative personality, AUT, and RAT on positive and negative *zhongyong* (including their interaction).

	**Creative personality**			**AUT**			**RAT**		
	**β**	***t***	***p***	**β**	***t***	***p***	**β**	***t***	***p***
Positive *zhongyong*	−0.003	−0.027	0.978	−0.103	−0.911	0.364	0.181	1.668	0.098
Negative *zhongyong*	−0.231	−2.368	0.020^*^	0.087	0.871	0.386	−0.103	−1.077	0.284
Interaction	0.110	1.115	0.267	−0.100	−0.988	0.325	−0.177	−1.829	0.070

* *p < 0.05*.

## Discussion

The results of Study 1 lent some support for our expected relationship but did not reach a statistically significant level. A possible reason for this is that the *zhongyong* personality scale that we compiled may not have revealed the participants' true *zhongyong* personality because of social desirability bias. For this reason, we used an implicit method to measure *zhongyong* in Study 2.

## Study 2

To minimize social desirability bias, we used the IAT method in Study 2 to assess implicit *zhongyong* personality and to check the findings of Study 1.

### Participants and Procedure

Before recruiting the participants, we again used G^*^Power 3.1 to estimate the minimum sample size suitable for a 2 × 2 between-subjects *F* test. According to Faul et al. (2007), when setting the effect size (*f*) at 0.25 (medium level), the error probability (α) at 0.05 (as a common practice), the power (1 – β) at 0.8 (as a common practice), the number of groups at 4, and the degree of freedom at 1, the calculated minimum sample size was 128.

In reality, another 144 undergraduate students (30 male, 114 female) aged between 18 and 28 (*M* = 19.62, *SD* = 1.49) from three classes of a university-wide selective course took part in the study in a classroom-based session. None of the participants had experience of taking tests similar to those used in our study. After they had consented to participate, the students were asked to complete the AUT, the RAT, and the creative personality scale in sequence using their mobile telephones on Wenjuanxing, a Chinese online questionnaire survey platform. They were asked to complete the *zhongyong* IAT using their mobile telephones on DiggMind, a behavioral experiment platform similar to E-prime but suitable for mobile devices. The total duration of these tests was ~30 mins.

### Methods

The AUT, the RAT, and the creative personality scale used in this study were identical to those used in Study 1. The *zhongyong* IAT was a modification of the self-esteem IAT (Greenwald and Farnham, 2000). During the first round, words denoting the concept of “self” (such as “me,” “my,” and “myself”) and the six positive *zhongyong* words used the same response button, while words associated with the concept of “other” (using words such as “them,” “their,” and “themself”) and the six negative *zhongyong* words shared a different response button. Following this, the categorization task was reversed: the self was grouped with negative *zhongyong* words, and the other with positive *zhongyong* words (see Table 4 for the complete procedure). In the case of incorrect responses, 600 ms of reaction time was added. If the self–positive pair in Block 4 took less time than the other–positive pair in Block 7, this was taken to indicate that the participant was implicitly demonstrating a positive *zhongyong* personality; if the self–negative pair in Block 7 took less time than the other–negative pair in Block 4, this was taken to indicate that the participant was implicitly demonstrating a negative *zhongyong* personality.

**Table 4:** Procedure for conducting the *zhongyong* IAT.

**Order of block**	**Number of trials**	**Function**	**Left button (number of trials)**	**Right button (number of trials)**
1	6	Practicing	Positive (3)	Negative (3)
2	6	Practicing	Self (3)	Other (3)
3	12	Practicing	Self (3) + positive (3)	Other (3) + negative (3)
4	24	Formal	Self (6) + positive (6)	Other (6) + negative (6)
5	6	Practicing	Negative (3)	Positive (3)
6	12	Practicing	Self (3) + negative (3)	Other (3) + positive (3)
7	24	Formal	Self (6) + negative (6)	Other (6) + positive (6)

### Results

Table 5 shows a cross-tabulation of the distribution of positive and negative *zhongyong* personality in the participants, as measured by the IAT. The results were consistent with those of Study 1: the majority of the participants demonstrated a positive *zhongyong* personality but not a negative *zhongyong* personality, while few of the participants possessed a negative *zhongyong* personality without also displaying a positive *zhongyong* personality. This again supports our conception of negative *zhongyong* as an excessive form of *zhongyong*, because only when one has first developed a positive *zhongyong* personality, can their *zhongyong* personality further reach the excessive amount manifesting in the form of negative *zhongyong*.

**Table 5:** The distribution of the number of participants who demonstrated implicit positive and negative *zhongyong* personality, as measured by the IAT.

		**Implicit**	**negative** ***zhongyong***		**Total**
			**No**	**Yes**	
Implicit	positive *zhongyong*	No	12	2	14
		Yes	113	17	130
	Total		125	19	144

**Figure 3:**
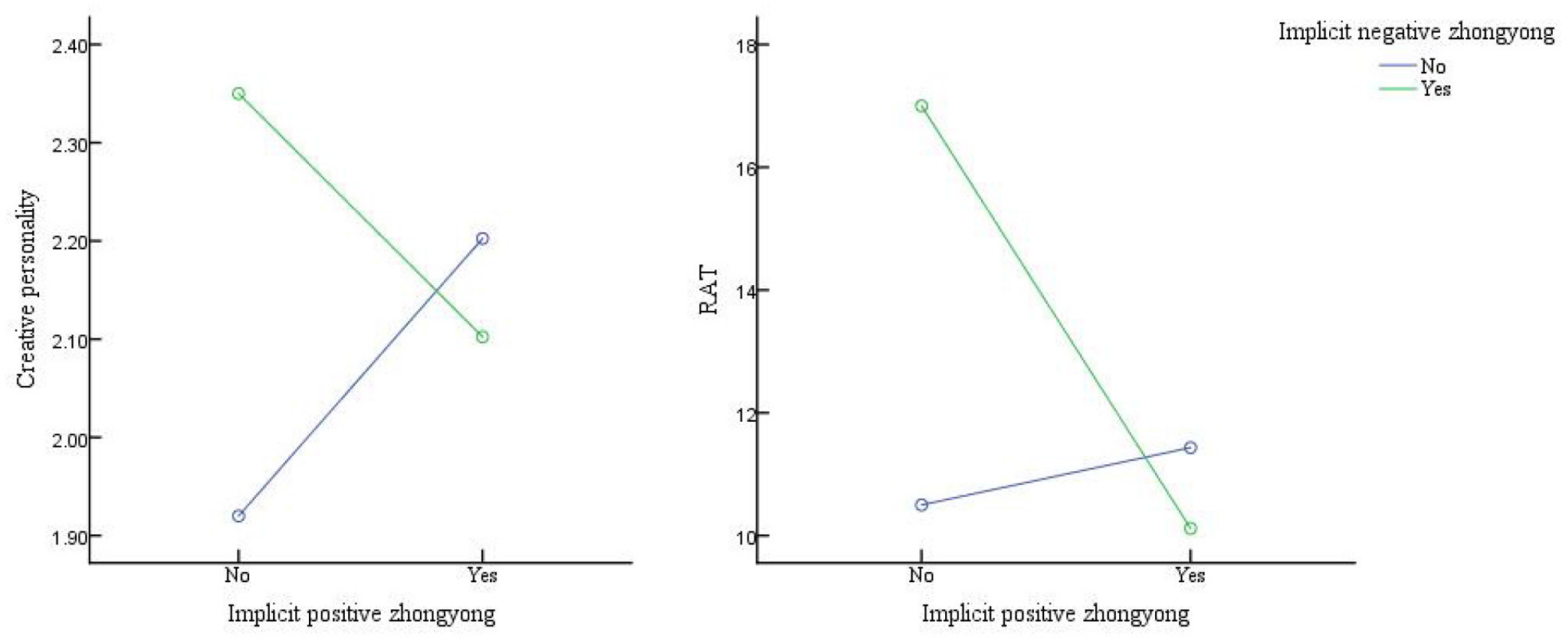
The simple effects of the interaction between implicit positive and negative *zhongyong* on creative personality (left) and RAT (right).

After homogeneity of variance was tested (*p*-values 0.756, 0.352, and 0.934 for creative personality, AUT, and RAT, respectively), three 2 (positive *zhongyong*: no/yes) × 2 (negative *zhongyong*: no/yes) non-repeated ANOVAs were computed for creative personality, AUT, and RAT. The results revealed two significant interactions between positive *zhongyong* and negative *zhongyong* in relation to creative personality and the RAT (see Table 6). We then analyzed the simple effects for both interactions (see Figure 3). This analysis further supported our prediction that for participants who did not have an implicit negative *zhongyong* personality, their implicit positive *zhongyong* personality would positively predict their creativity, while for participants with an implicit negative *zhongyong* personality, their positive *zhongyong* personality would negatively predict their creativity.

**Table 6:** The results of analyses of variance for the effects of positive and negative *zhongyong* as well as their interation on creative personality, AUT, and RAT.

	Creative personality				AUT				RAT			
	***F***	***df***	***p***	** $\eta_{\text{p}}^{2}$ **	***F***	***df***	***p***	** $\eta_{\text{p}}^{2}$ **	***F***	***df***	***p***	** $\eta_{\text{p}}^{2}$ **
Positive *zhongyong*	0.03	1	0.858	0.00	0.97	1	0.326	0.01	5.13	1	0.025^*^	0.04
Negative *zhongyong*	2.90	1	0.091	0.02	2.04	1	0.155	0.01	3.89	1	0.050^*^	0.03
Interaction	7.49	1	0.007^**^	0.05	0.92	1	0.340	0.01	8.85	1	0.003^**^	0.06

* *p < 0.05*;

** *p < 0.01*.

### Discussion

Based on the results of Studies 1 and 2, we can conclude that negative *zhongyong* is an excessive form of *zhongyong* that inhibits creativity. We can also conclude that only a moderate level of *zhongyong* is conducive to creativity, with both deficiency and excess being harmful. This interaction effect was found for creative personality and the RAT but not for the AUT, which is consistent with previous findings (Zhou et al., 2019, 2021; Zhang et al., 2020). This implies that the RAT and *zhongyong* thinking have a similar cognitive mechanism, but the AUT does not. Zhu et al. (2019) found a threshold-setting effect of convergent thinking; that is, only when convergent thinking capacity reached a certain level did divergent thinking begin to play a role in scientific creativity. This suggests that *zhongyong* thinking may be important to scientific creativity.

## General Discussion

In this paper we present the results of two empirical studies conducted to examine the non-linear relationship between *zhongyong* and creativity. Based on these studies, we conclude that excessive *zhongyong* is detrimental to creativity and moderate *zhongyong* is conducive to creativity. These findings shed light on the previously inconsistent findings regarding the relationship between *zhongyong* and creativity.

Our study has two major implications. First, our work highlights the possibility of non-linear relationships between constructs. Both *zhongyong* in China and the doctrine of the mean in Europe emphasize moderation, which means that the relationship between positive antecedents and ideal consequences is not necessarily monotonic and that the “more is better” attitude may be misguided. The “too much of a good thing” effect, i.e., the inverse U-shaped relationship, has aroused more and more attention. In the field of psychology, this effect has been observed in relation to individual personality traits (e.g., Bozionelos et al., 2014; Nieß and Biemann, 2014; Vergauwe et al., 2018), skills (e.g., Zettler and Lang, 2015), and demographic variables such as age (e.g., von den Driesch et al., 2015) and family socioeconomic status (e.g., Ren and Xin, 2013). In the fields of economics and management, researchers have also found this effect in resource ownership (e.g., Rotolo and Messeni Petruzzelli, 2013; Shao et al., 2013; Ren and Chadee, 2017; Fisman et al., 2020), positive and negative work experience (e.g., Carette et al., 2013; Lee et al., 2013; Stouten et al., 2013; Lam et al., 2014; Rapp et al., 2014; Astakhova, 2015; Burnett et al., 2015; Zhang and Long, 2016; Mo et al., 2019), employee autonomy (e.g., Lee et al., 2017), emotional expression rules (e.g., Christoforou and Ashforth, 2015), and group diversity (e.g., Ali et al., 2014; Wei et al., 2015; Vicentini and Boccardelli, 2016; Dayan et al., 2017). These studies adopted a new perspective of curvilinear relationships or interaction effects to revisit debates in previously published literature, gaining new insights either (a) by identifying an inflection point after which the positive effects turned negative as a result of breaking up the balance between gains and losses, or (b) by introducing a new factor as a moderator, where the product of the independent variable and the moderating variable was instrumental in influencing the dependent variable (for reviews, see Grant and Schwartz, 2011; Pierce and Aguinis, 2013; Haans et al., 2016; Xing et al., 2018). The study of *zhongyong* itself is no exception. The inconsistency in previous findings occurred because scholars did not adopt *zhongyong* thinking; they failed to use a *zhongyong* approach to understand *zhongyong* itself. The present study helps us to understand the positive and negative sides of *zhongyong* and to realize that a moderate level of *zhongyong* is conducive to stimulating creativity, while too much or too little is useless.

Second, we argue for a reconceptualization of creativity in Chinese culture. In the past, Chinese people have been labeled “not creative,” partly because the modern industrial and scientific revolutions did not originate in China. Many scholars have tried to determine the cultural reasons for this lack of creativity, writing books with titles such as *Why Asians Are Less Creative Than Westerners* (Ng, 2001) and *Liberating the Creative Spirit in Asian Students* (Ng, 2004). These books have proposed that *zhongyong* thinking, collectivism, hierarchy, obedience to authority, self-inhibition, and mechanical learning in Confucian cultures hinder the development of creativity. Sometimes even Chinese people themselves are not confident in their creativity. They may question, criticize, or even completely deny long-established Confucian ideas and collectivist values in Chinese traditional culture, believing that they inhibit the development of creativity. However, as our understanding of creativity increases, the incompleteness of these views becomes apparent. Creative thinking includes both divergent thinking and convergent thinking (Guilford, 1967) and creative products should be novel and practical (Mayer, 1999). Innovation is not only in the minds of individuals but also depends on collaboration within groups and sometimes even the cooperation of a whole society (Simonton and Ting, 2010). Several studies have found that Chinese culture is negatively correlated with some aspects of creativity, such as divergent thinking (Kim et al., 2011), product novelty (Hofstede, 2001), and individual independence (De Dreu, 2010). However, Chinese culture has been shown to promote convergent thinking (e.g., Cheung et al., 2016), product practicality (Xie and Paik, 2019), and success in epidemic prevention and control, poverty reduction, and environmental management through large-scale collective action (Han and Huang, 2018). It can be said that creativity in Chinese culture manifests differently, incorporating wisdom that has unique Chinese characteristics but is also of universal value. We must adopt a neutral, objective perspective to re-assess creativity in Chinese culture. *Zhongyong* is at the core of Chinese culture, and its relationship with creativity is of particular research interest. Miron-Spektor and Erez (2017) discussed the inherently paradoxical nature of creativity from various angles, including that of the coexistence of novelty and practicality in a creative outcome, and this suggests that the essence of *zhongyong* shares aspects of the essence of creativity. We should not only cease to regard *zhongyong* as the opposite of creativity but also dig more deeply into the unique wisdom that *zhongyong* contributes to problem-solving.

Our study has several limitations. First, we were unable to measure positive and negative *zhongyong* on one scale to find an inflection point between positive and negative effects in continuous data. This should be addressed in future research. Second, in Study 2, the small numbers of participants in some of the groups (the non-positive and non-negative *zhongyong* group and the both positive and negative *zhongyong* group) may have led to some statistical bias. However, the non-significant result of the variance homogeneity test indicated that the ANOVA results were basically acceptable.

In summary, the non-linear relationship between *zhongyong* and creativity uncovered by our research sheds light on the inconsistent findings of previous studies. Our results contribute to a more comprehensive understanding of *zhongyong* and offer clear insights into creativity from an in-depth cultural perspective.

## Data Availability Statement

The datasets presented in this article are not readily available because the data that support the findings of this study are available on request from the corresponding author. The data are not publicly available due to privacy or ethical restrictions. Requests to access the datasets should be directed to molei@m.scnu.edu.cn.

## Ethics Statement

The studies involving human participants were reviewed and approved by Human Research Ethics Committee for Non-Clinical Faculties in School of Psychology, South China Normal University. The patients/participants provided their written informed consent to participate in this study.

## Author Contributions

All authors listed have made a substantial, direct, and intellectual contribution to the work and approved it for publication.

## Funding

This work was supported by grants from the National Social Science Foundation of China (19ZDA360, 19BKS162), the Key Institute of Humanities and Social Science in the MOE of China (16JJD880025), Major Basic Research and Applied Research Projects of the Guangdong Education Department (#2017WZDXM004), and the Major Project of Social Science in South China Normal University (ZDPY2208).

## Conflict of Interest

The authors declare that the research was conducted in the absence of any commercial or financial relationships that could be construed as a potential conflict of interest.

## Publisher's Note

All claims expressed in this article are solely those of the authors and do not necessarily represent those of their affiliated organizations, or those of the publisher, the editors and the reviewers. Any product that may be evaluated in this article, or claim that may be made by its manufacturer, is not guaranteed or endorsed by the publisher.
